# 889. Clinical and Economic Outcomes of Autologous Skin Grafts: A Retrospective Claims Database Analysis

**DOI:** 10.1093/jbcr/irag033.375

**Published:** 2026-04-06

**Authors:** Roselle E Crombie, Lauren A Do, Mihail Samnaliev, Matthew Sussman, Lisa J Gould

**Affiliations:** Connecticut Burn Center/Yale New Haven Health, Westport, CT; Stratevi, LLC, Boston, MA; Stratevi, LLC, Boston, MA; Stratevi, LLC, Boston, MA; South Shore Health, Warwick, RI

## Abstract

**Introduction:**

Nonthermal full-thickness wounds (NFTW) resulting from trauma or surgery are complex wounds requiring intensive management. Autologous skin grafting is often required for definitive closure. However, comprehensive evidence describing the clinical and economic burden of patients undergoing skin grafts is limited.

**Methods:**

A retrospective cohort analysis was performed utilizing the Clarivate’s Real-world Data Repository® national claims database between 01/01/2019 and 08/31/2024. Patients aged ≥15 years with a primary NFTW diagnosis resulting from traumatic, surgical, or other select soft tissue wounds were indexed on a subsequent skin graft procedure. Separate cohorts were constructed and analyzed according to the care setting in which the index procedure was performed. The Kaplan–Meier method was used to assess 90-day post-index adverse outcome rates and select adjunct procedures and medication use. All-cause healthcare resource utilization (HRU) and costs (expressed in 2024 US dollars) were estimated over 12 months post-index.

**Results:**

The study included 26 117 patients with a NFTW. Mean age was 59.3 years and 43.3% were female. The sample exhibited substantial medical complexity with multiple comorbidities: 41.2% were smokers, 32.0% were obese, 31.8% had diabetes or diabetes-related comorbidities, and 21.5% were malnourished. Drug or alcohol abuse (15.6%), frailty (13.6%), and autoimmune diseases/immunodeficiency (10.4%) were also common. Wound etiology was diverse, with traumatic wounds accounting for 56.1% and surgical wounds for 29.7% of cases. Within 90 days, the inpatient cohort had the highest rates of adverse outcomes: 7.6% required regrafting and 4.9% experienced graft failure. Total mean annual costs were highest in the inpatient cohort ($266 231, standard deviation [SD] = $458484), followed by outpatient hospital ($62 619, SD = $132510) and office/clinic ($14 261, SD = 36 894) cohorts with an average all-cohort cost of $128 959 (SD = $306485).

**Conclusions:**

Individuals receiving skin grafts for NFTW are medically complex with high rates of complications and costs, highlighting the need for novel interventions that may reduce the clinical and economic burden.

**Applicability of Research to Practice:**

Results may inform models of the economic value of emerging treatments and guide strategies to optimize post-skin graft care, minimize complications, and reduce payer burden.

**Funding for the study:**

This study was funded by AVITA Medical. Acquisition of the database was funded by AVITA Medical. Lead author, in the spirit of academics and belief in the project, was not compensated for this project.

